# Bronchiolitis and bacteraemia caused by *Burkholderia gladioli* in a non-lung transplantation patient

**DOI:** 10.1002/nmi2.64

**Published:** 2014-09-25

**Authors:** O Imataki, N Kita, H Nakayama-Imaohji, J-i Kida, T Kuwahara, M Uemura

**Affiliations:** 1 Division of Haematology and Stem Cell Transplantation, Department of Internal Medicine, Faculty of Medicine, Kagawa UniversityKagawa, Japan; 2 Division of Respiratory Medicine, Department of Internal Medicine, Faculty of Medicine, Kagawa UniversityKagawa, Japan; 3 Division of Molecular Microbiology, Faculty of Medicine, Kagawa UniversityKagawa, Japan

**Keywords:** *Burkholderia gladioli*, chronic lower respiratory infection, diffuse panbronchiolitis, immunocompromised host, non-fermenting Gram-negative bacillus, non-lung transplantation patient

Dear Editor,

The Gram-negative bacillus *Burkholderia gladioli* is known to be a causative pathogen of pneumonia in cystic fibrosis 1,2. Chronic lower respiratory infections involving *B. gladioli* are associated with poor graft survival and unfavourable outcomes 1,2. *Burkholderia gladioli* was first identified as a plant pathogen in gladiolus, iris and rice, but this bacterium is found in diverse environments including soil, plants and the human respiratory tract. Its pathogenesis in humans was first described in the mid-1990s in case reports of cystic fibrosis 3 and chronic granulomatous disease 4, and the majority of the reported *B. gladioli* infections have been in immunocompromised adults, e.g. human immunodeficiency virus-infected patients, and newborns 5. Although it is still a fairly uncommon pathogen, *B. gladioli* in humans is an opportunistic pathogen and is associated with a poor prognosis.

**Figure 1:**
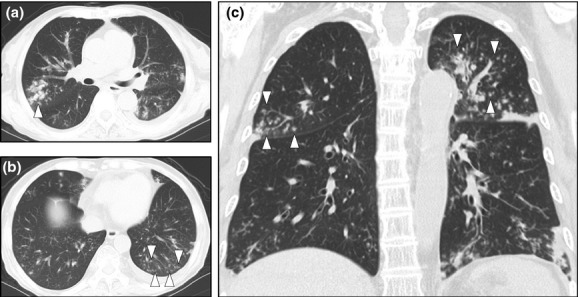
Pulmonary infection by *Burkholderia gladioli* presenting as diffuse panbronchiolitis. (a) Transverse view showing the transbronchial distribution of small opaque nodules in the right upper lobe (S2), indicated by arrowheads. (b) Nodular infiltration shadows formed a consolidation in the left lower lobe (S10), indicated in the area surrounded by arrowheads. (c) The coronal image construction shows diffuse distribution of airway thickness located in multiple lobes, similar to diffuse panbronchiolitis, marked by arrowheads.

The genus *Burkholderia* was formerly classified as the genus *Pseudomonas*. The virulence of *B. gladioli* in humans is recognized to be low, and the clinical manifestations of this pathogen in non-lung transplantation patients have not been fully elucidated. We treated a 62-year-old female patient with myasthenia gravis, a thymoma and immune-mediated granulocytopenia. She had been treated with tacrolimus for the previous 12 months. From her sputum, non-fermenting Gram-negative rods were cultured microbiologically. The isolate was oxidase-negative, indole-negative and nitrate-negative. The 16S rRNA detection in our collaborator's laboratory confirmed *B. gladioli* as a 99% comparable DNA sequence. The patient's case was complicated with bronchiolitis followed by bacteraemia due to *B. gladioli*. The infection of *B. gladioli* presented as diffuse panbronchiolitis (Fig.1) and progressed into repeated bacteraemia. The pathogen was not isolated because a laboratory technician made an arbitrary decision not to proceed further to determine the species of the causative organism, although the patient had been suffering from repeated bacteraemia due to the same bacillus. Our institute's laboratory reported the pathogen only as ‘an environmental non-fermenting Gram-negative rod.’

Non-fermenting Gram-negative bacilli are bradytrophic and can survive for long periods in a wet environment without sufficient nutrients. Such bacilli include *Pseudomonas* spp., *Acinetobacter* spp., *Stenotrophomonas* spp., *Chryseobacterium* spp. and *Burkholderia* spp., all of which are opportunistic pathogens. Non-fermenting Gram-negative bacilli have the following microbiological characteristics: (1) they produce various β-lactamases in nature, (2) they show multidrug resistance in nature, (3) they easily acquire plasmids harbouring antibiotic-resistant genes, and (4) they form a biofilm on surfaces to colonize. All of these features are linked to clinical issues in the treatment of infection, and they are linked to unfavourable outcomes.

As stated in the position paper distributed by the US Health Care Infection Control Practices Advisory Committee (HICPAC), the surveillance of newly emerging pathogens is a critically important component of prudent infection control. Microbiological identification is also crucial, especially using samples obtained in clinical settings. In our patient, the isolated bacterium was not multidrug-resistant, fortunately; she was treated successfully with a carbapenem antibiotic, meropenem (0.5 g, three times per day, administered for 11 days). We recommend the microbiological identification of non-fermenting Gram-negative bacilli according to the clinical situation, even when the bacillus is likely to be found in the environment and is an unremarkable microbe. To do so, interdisciplinary interactions between clinical and laboratory personnel are helpful. The value of medical microbiology is based on interactive laboratories.
